# Evaluating Pain Management Competency in Dementia via Virtual Simulations: Research Implications

**DOI:** 10.1093/geroni/igaf122.3372

**Published:** 2025-12-31

**Authors:** Yo-Jen Liao, Ying-Ling Jao, Marie Boltz, Nai-Ching Chi, Diane Berish, Terrence Murphy

**Affiliations:** The Pennsylvania State University, University Park, Pennsylvania, United States; The Pennsylvania State University, University Park, Pennsylvania, United States; The Pennsylvania State University, University Park, Pennsylvania, United States; University of Iowa, Iowa City, Iowa, United States; The Pennsylvania State University, University Park, Pennsylvania, United States; The Pennsylvania State University, University Park, Pennsylvania, United States

## Abstract

Pain under-recognition in dementia highlights the importance of clinicians’ pain management competencies in improving care for this population. Virtual simulation presents a potentially more efficient alternative to direct observation for competency evaluation. Simulation allows clinicians to review patient histories, ask questions, perform assessments, and administer treatments on mock cases with interactive responses based on pre-designed scenarios. However, virtual simulation has not been extensively used in dementia pain management research. This study reports the challenges and strategies in using virtual simulations to evaluate nurses’ pain management competencies for nursing home residents with dementia. Three scenarios, each representing different dementia stages and pain characteristics, were developed in collaboration with a technology company. The competency evaluation involves two steps. First, after viewing an orientation video, nurses performed pain management on two randomly assigned scenarios. Second, raters reviewed the simulation reports and assessed competencies using the Pain Competency Evaluation in Dementia Scale. Seventeen nurses/nursing students participated, with an average age of 25 years (94% female) and pain competency score of 66%. Challenges included participating nurses improperly exiting the simulation, resulting in unsaved reports. Raters encountered errors in simulation reports. These issues were addressed with the technology company. Scenario development took five months, involving scripting, validation, and refinement. Limitations included difficulties in showing subtle facial expressions and word limits in medical records. Subtle expressions were converted into words, and text was condensed in medical records. Future research should consider the benefits and limitations of virtual simulation while ensuring the design is optimized for research objectives.

